# Supplementary material for: p53 and p21 dynamics encode single-cell DNA damage levels, fine-tuning proliferation and shaping population heterogeneity
Source: Commun Biol. 2023 Nov 24;6:1196. doi: 10.1038/s42003-023-05585-5 (PMC10673849; doi:10.1038/s42003-023-05585-5)
Supplement: Supplementary file 2 — Supplementary Information [file 42003_2023_5585_MOESM2_ESM.pdf]

Figure 1S:

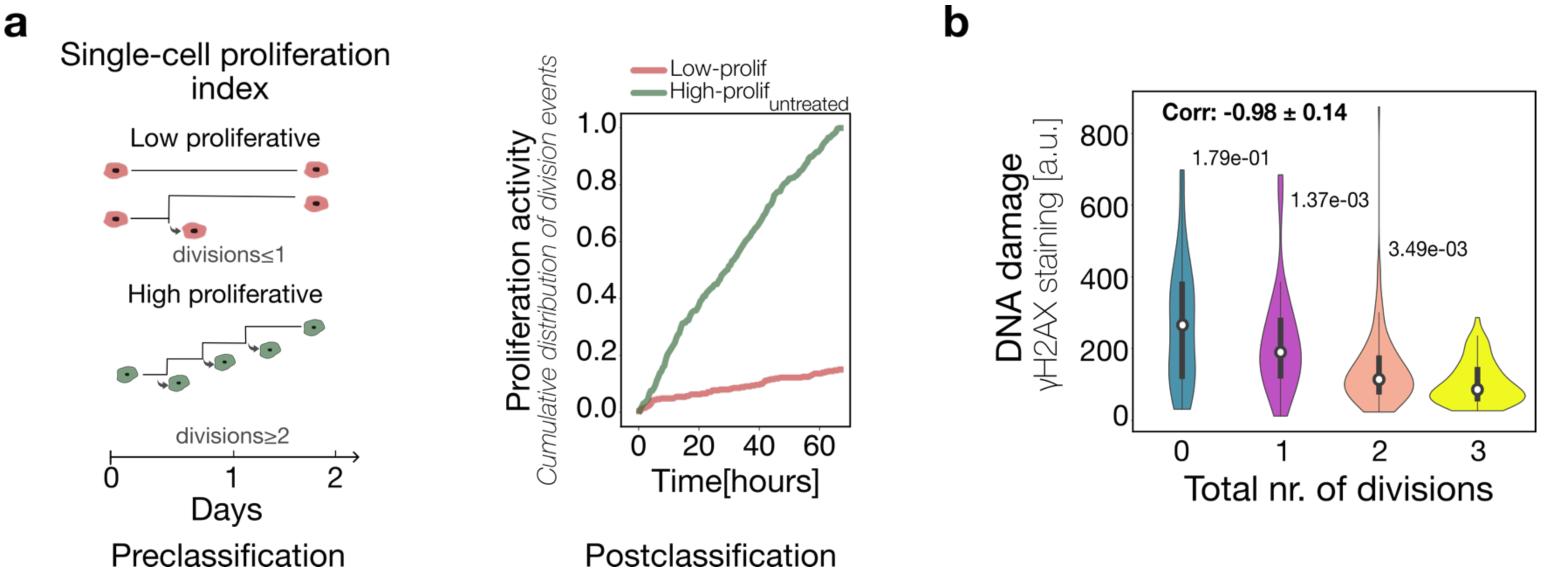

a) Left: a sketch of the definition of low (up to 1 division) and high (more than 2 divisions) proliferative cells for a time frame of 2 days. We used the first 2 days to classify the cells into two groups according to their total number of divisions in this time-lapse. Next, we used this classification to determine the proliferation activity, see the plot from the right: the cumulative distribution of division events for low and high proliferative cells.

b) Violin plot of the DNA damage levels classified by the total number of divisions, with the corresponding p-values and correlation coefficients.

Figure 2S:

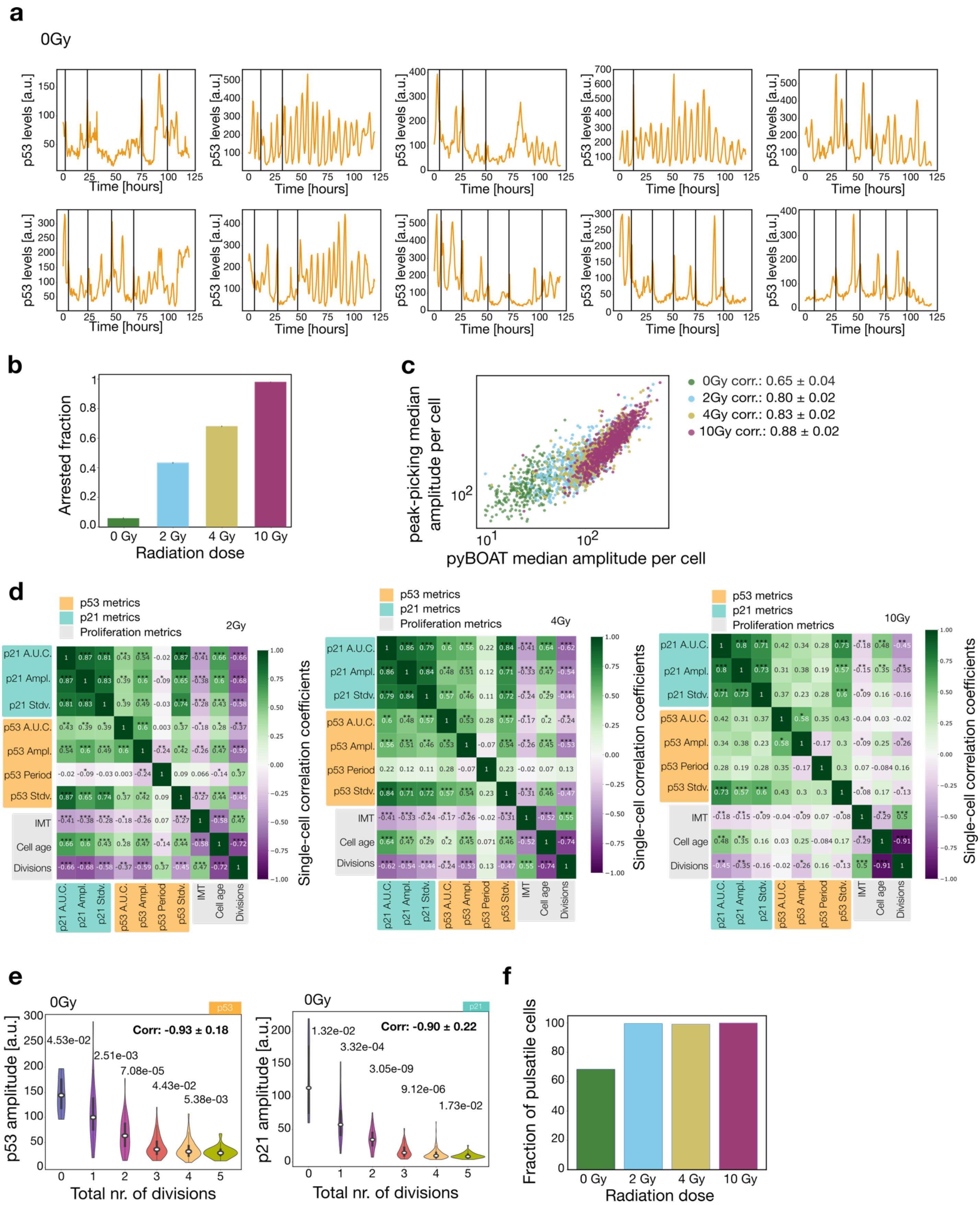
a
0Gy
p53 levels [a.u.]
Time [hours]
b
Arrested fraction
0 Gy
2 Gy
4 Gy
10 Gy
Radiation dose
c
peak-picking median amplitude per cell
pyBOAT median amplitude per cell
0Gy corr.: 0.65 ± 0.04
2Gy corr.: 0.80 ± 0.02
4Gy corr.: 0.83 ± 0.02
10Gy corr.: 0.88 ± 0.02
d
p53 metrics
p21 metrics
Proliferation metrics
2Gy
4Gy
10Gy
p21 A.U.C.
p21 Ampl.
p21 Stdv.
p53 A.U.C.
p53 Ampl.
p53 Period
p53 Stdv.
IMT
Cell age
Divisions
Single-cell correlation coefficients
e
0Gy
p53 amplitude [a.u.]
Corr: -0.93 ± 0.18
4.53e-02
2.51e-03
7.08e-05
4.43e-02
5.38e-03
Total nr. of divisions
p21 amplitude [a.u.]
Corr: -0.90 ± 0.22
1.32e-02
3.32e-04
3.05e-09
9.12e-06
1.73e-02
f
Fraction of pulsatile cells
Radiation dose

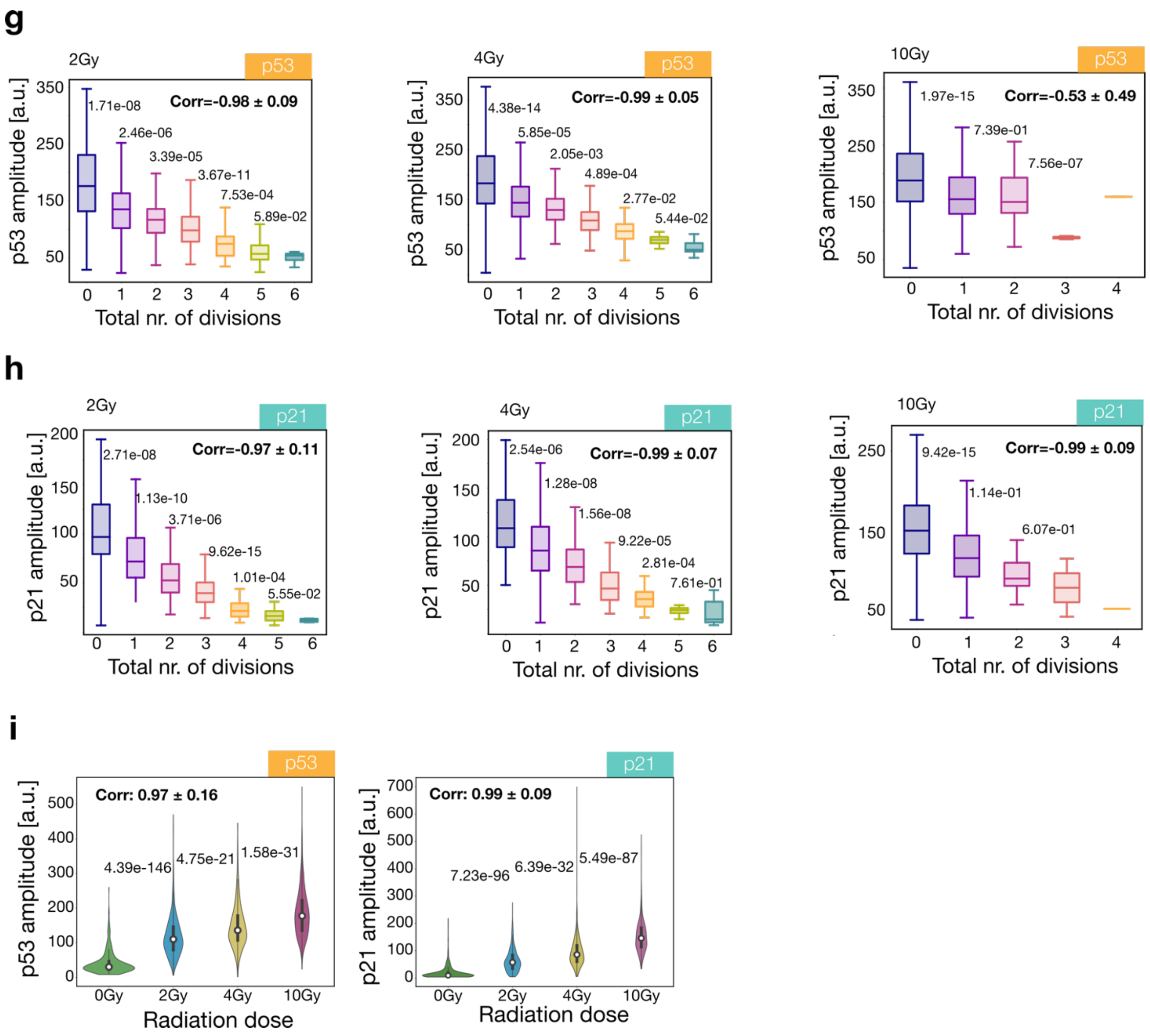

a) Examples of p53 single cell traces of the untreated condition (0 Gy) with the corresponding division times (vertical lines).

b) Bar plot with the fraction of arrested cells for each radiation dose.

c) Scatter plot showing the median values of amplitude per cell acquired through two distinct methods (continuous wavelet transform with pyBOAT and peak picking) for each condition with different colors in a logarithmic scale for both axes, accompanied by the respective correlation coefficient calculated through the Pearson correlation.

d) Heatmap of the mean single-cell correlation coefficients of p53 (yellow), p21 (green), and proliferation metrics (grey) for different radiation doses (from left to right: 2, 4, and 10 Gy). A positive high correlation is shown in green and a negative correlation in purple. The correlation coefficients are shown in the respective boxes.

e) Violin plot of p53 (left) and p21 amplitude (right) of the untreated condition clustered by the total number of divisions with the corresponding p-values and correlation coefficients.

f) Bar plot with the fraction of pulsatile cells for each radiation dose.

g) Boxplot of p53 amplitude for different radiation doses with the corresponding p-values and correlation coefficients (see Table1S for more p-values).
h) Boxplot of p21 amplitude for different radiation doses with the corresponding p-values and correlation coefficients (see Table2S for more p-values).
i) Violin plot of p53 (left) and p21 amplitude (right) for different radiation doses with the corresponding p-values and correlation coefficients.

Figure 3S:

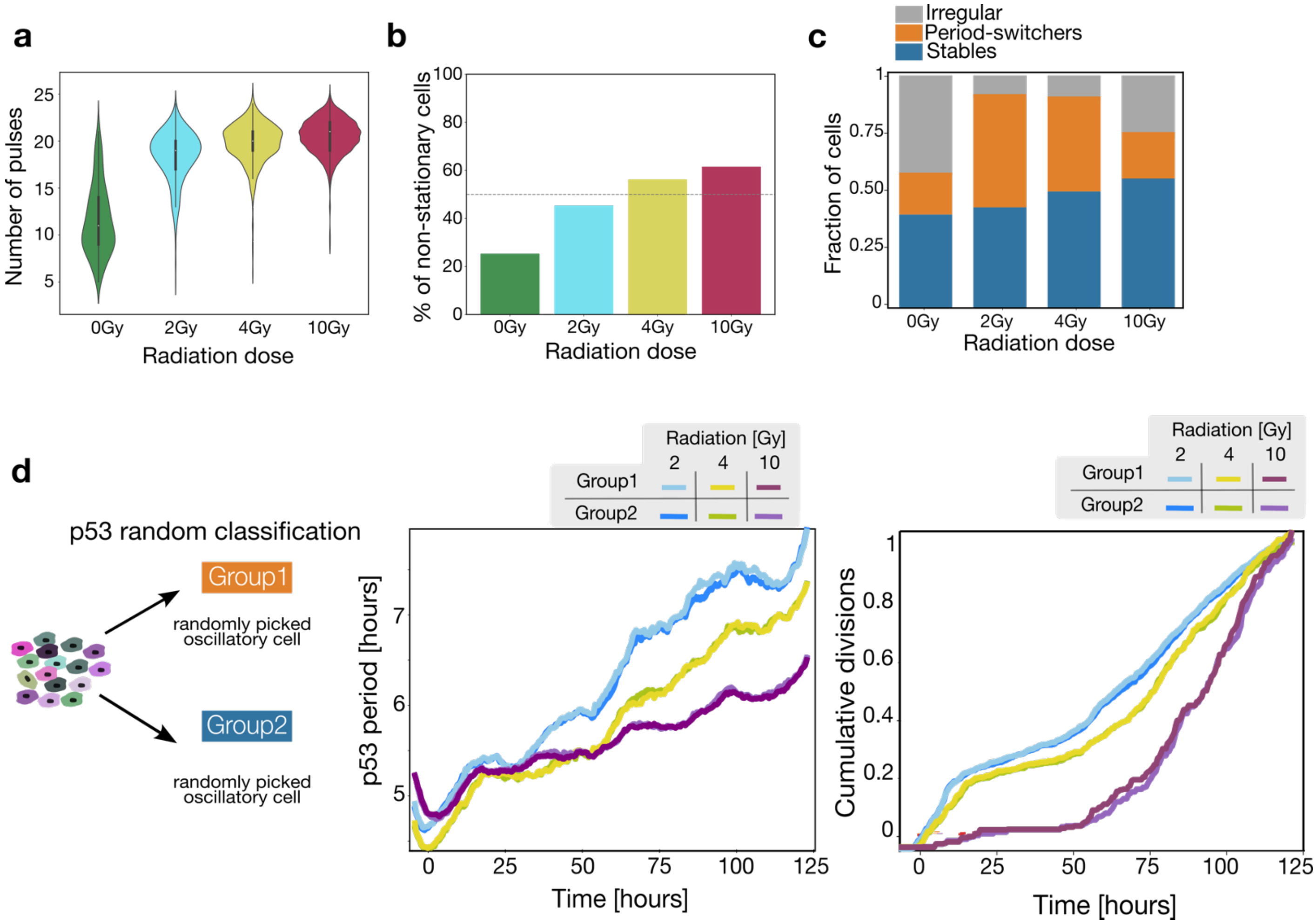

a) Violin plot of the number of pulses per cell of each radiation dose computed by peak picking.
b) Fraction of non-stationary cells for each radiation dose computed using the Augmented Dickey Fuller test. This test gives us a p-value that we use to analyze the stationarity of a particular signal. Here, we show the fraction of cells that had a p-value higher than 0.05, which were considered as non-stationary.
c) Fraction of cells from each period group (stables, prolongers, or irregulars) for each particular condition.
d) Sketch of p53 random classification in two groups (left plot). In the middle plot, we see the median p53 period of each group of cells corresponding to the random classification. In the final plot, we have the cumulative distribution of division events for cells within each group according to the random classification.

Table 1S: p-values to Fig.2Sg

2Gy

#divisions	1	2	3	4	5	6
**0**	1,71E-08	6,72E-14	1,66E-19	1,67E-28	1,53E-32	6,17E-23
**1**	0	2,46E-06	1,86E-18	2,66E-44	2,27E-38	9,40E-09
**2**	2,46E-06	0	3,39E-05	6,36E-24	3,34E-27	3,22E-08
**3**	1,86E-18	3,39E-05	0	3,67E-11	4,00E-17	7,19E-07
**4**	2,66E-44	6,36E-24	3,67E-11	0	7,50E-04	5,40E-04
**5**	2,27E-38	3,34E-27	4,00E-17	7,50E-04	0	5,90E-02
**6**	9,40E-09	3,22E-08	7,19E-07	5,40E-04	5,90E-02	0

4Gy

#divisions	1	2	3	4	5	6
**0**	4,38E-14	8,58E-25	2,55E-20	1,74E-26	1,23E-25	7,47E-12
**1**	0	5,85E-05	1,07E-07	1,69E-14	7,78E-15	9,61E-08
**2**	5,85E-05	0	2,10E-03	2,43E-10	6,58E-12	8,37E-07
**3**	1,07E-07	2,10E-03	0	4,90E-04	3,02E-07	9,64E-07
**4**	1,69E-14	2,43E-10	4,90E-04	0	2,80E-02	1,10E-03
**5**	7,78E-15	6,58E-12	3,02E-07	2,80E-02	0	5,40E-02
**6**	9,61E-08	8,37E-07	9,64E-07	1,10E-03	5,40E-02	0

10Gy

#divisions	1	2	3	4
**0**	1,97E-15	5,90E-04	1,96E-05	-
**1**	0	7,40E-01	2,80E-06	-
**2**	7,40E-01	0	7,56E-07	-
**3**	2,80E-06	7,56E-07	0	-
**4**	-	-	-	0

Table 2S: p-values to Fig.2Sh

2Gy

#divisions	1	2	3	4	5	6
**0**	2,71E-08	8,41E-18	2,40E-27	3,51E-36	7,24E-39	1,90E-36
**1**	0	1,23E-10	2,85E-39	5,32E-85	1,23E-93	1,88E-19
**2**	1,23E-10	0	3,71E-06	2,50E-23	1,58E-30	4,16E-20
**3**	2,85E-39	3,71E-06	0	9,62E-15	4,04E-24	3,65E-11
**4**	5,32E-85	2,50E-23	9,62E-15	0	1,00E-04	2,00E-04
**5**	1,23E-93	1,58E-30	4,04E-24	1,00E-04	0	5,50E-02
**6**	1,88E-19	4,16E-20	3,65E-11	2,00E-04	5,50E-02	0

4Gy

#divisions	1	2	3	4	5	6
**0**	2,54E-06	2,30E-28	8,56E-35	6,23E-42	1,15E-54	2,40E-07
**1**	0	1,28E-08	2,46E-21	3,10E-33	2,93E-47	4,77E-07
**2**	1,28E-08	0	1,56E-08	9,77E-19	3,27E-29	2,94E-05

**3**	2,46E-21	1,56E-08	0	9,22E-05	4,96E-11	1,60E-03
**4**	3,10E-33	9,77E-19	9,22E-05	0	2,80E-04	6,40E-02
**5**	2,93E-47	3,27E-29	4,96E-11	2,80E-04	0	7,60E-01
**6**	4,77E-07	2,94E-05	1,60E-03	6,40E-02	7,60E-01	0

10Gy

#divisions	1	2	3	4
**0**	9,42E-15	6,42E-05	2,8E-01	-
**1**	0	1,14E-01	4,40E-01	-
**2**	1,14E-01	0	7,56E-07	-
**3**	4,40E-01	7,56E-07	0	-
**4**	-	-	-	0
